# Material Testing of Historic Bricks and Mortars in Degraded Masonry Structures

**DOI:** 10.3390/ma17133192

**Published:** 2024-06-29

**Authors:** Dariusz Bajno, Krzysztof Schabowicz, Agnieszka Grzybowska

**Affiliations:** 1 Department of Building Engineering, Faculty of Civil Engineering, Wroclaw University of Science and Technology, 50-370 Wrocław, Poland; dariusz.bajno@pwr.edu.pl (D.B.); krzysztof.schabowicz@pwr.edu.pl (K.S.); 2 Department of Building Structure, Faculty of Civil and Environmental Engineering and Architecture, Bydgoszcz University of Science and Technology, 85-796 Bydgoszcz, Poland

**Keywords:** permanent ruin, nondestructive testing, mycological research, scanning, masonry structures, structural and material protection

## Abstract

The subject of this article is material research carried out on the ruins of a medieval castle located in west-central Poland. This facility was built at the beginning of the 15th century by the Order of St. John, and during its long life, it was subjected to many reconstructions. Unfortunately, in 1975, it was destroyed by fire. Since then, it has been left in a state of advanced ruin, exposed to climatic influences without any protection. The subject of the research was to assess the possibility of maintaining such buildings in a severely degraded condition while ensuring their technical efficiency. The article discusses a particular instance of “consolidation” applied to a structure in a state of historical, architectural, and structural ruin. After the diagnosis, it was determined that the structure should be safeguarded using a minimally invasive method. The purpose of these activities was to answer the question of whether the structure could be left to continue operating despite failing to meet the requirements of current standards and regulations while posing an additional danger to itself and the environment,. This goal was achieved by obtaining a considerable amount of data on the condition of the materials embedded in the masonry structure, thanks to which the initial parameters for conducting an assessment of the technical condition of the damaged masonry structure and evaluating the degree of its danger were developed. The results of the research and analysis carried out and described in this article can be used in other similar situations where saving national heritage objects through “artificial modern” strengthening will be unsafe and will lead to a loss of their authenticity. We still have a long way to go to develop a comprehensive method for “in situ” diagnosis of heterogeneous masonry structures, so we should use possible techniques and knowledge to conduct such assessments and propose rescue methods for historically valuable objects in a way that could minimize the damage and that can “easily” disappear from our surroundings. Each study should have a specific purpose, not only research but also a long-term perspective, making it possible to leave material for further research and analysis, including testing new research methods in real conditions of its installation.

## 1. Introduction

Building ceramics is one of the oldest construction products [1,2]. Ordinary ceramic bricks were produced in Egypt before 4000 BC, while colored ceramic bricks were produced around 3000 BC. In Europe and Poland, the first stone and brick structures were built from hand-formed bricks (the so-called finger bricks) already in the 10th century. The mechanized production process began in the 18th century, while fully mechanized industrial production involving the use of a ring furnace and a mechanical press took place in the second half of the 19th century. The brick firing process takes place at temperatures of 800 to approximately 1200 °C and over 1500 °C in the case of refractory ceramics. Signs informing about their producers have been embossed on bricks since ancient times, and this principle is also used today. The basic building materials of most historical buildings were wood, stone, ceramics, and lime. While the technical condition of, e.g., wooden structures can be assessed quite accurately on the basis of dedicated acoustic and resistographic methods (measuring cutting resistance) among others, the same research techniques will not necessarily work in the assessment of masonry structures. Research on diagnostic methods for walls, including historical ones, is still at the stage of tests and analyses both in situ and in laboratories. So far, no effective method for assessing these structures has been developed [3,4,5,6,7,8,9,10,11]. The relationship between the history of the object and, especially, its last stage, when it was subjected to loads not expected of it, is close because it is the basis for the genesis of its technical wear and tear. This is not just about laboratory tests, for which it is possible to collect and destroy any number of samples. In the case under study, we are dealing with material built into a large-scale facility. Here, the results of possible tests as well as our knowledge and well-made decisions will determine the safety of the facility left for further operation.

## 2. Case Study

Each building structure is subject to aging processes, i.e., it wears out technically, environmentally, and functionally [1,12,13,14]. The pace of such processes depends on the awareness and care of these structures, as well as on the technology used to make them, external influences, and random events. Mainly, attention to the condition of buildings and structures will determine their technical and operational life [15]. The process of technical wear and tear of each building begins at the commencement of construction works and will always have a rapidly progressing tendency unless it is slowed down by appropriate handling, including systematically diagnosing the technical condition and allowing only justified changes in its structure, supported by appropriate analyses and computational simulations [1,12,13,16,17].

**Figure 1:**
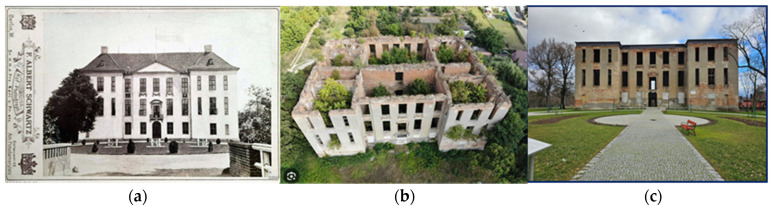
View of the castle: (**a**) in 1896 [21], (**b**) in 2018 [22], and (**c**) in 2023 (photo by authors).

**Figure 2:**
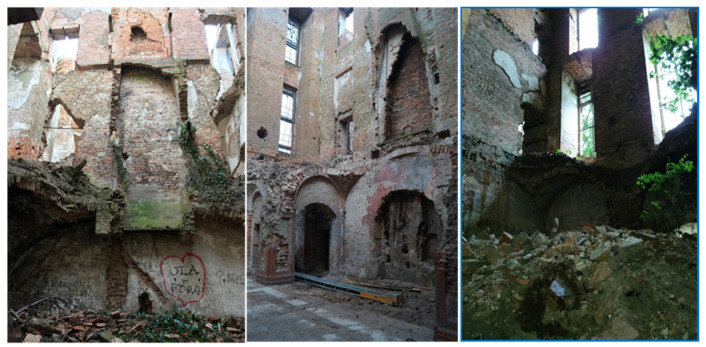
View of walls and vaults—as of 2018 (photos by authors).

**Figure 3:**
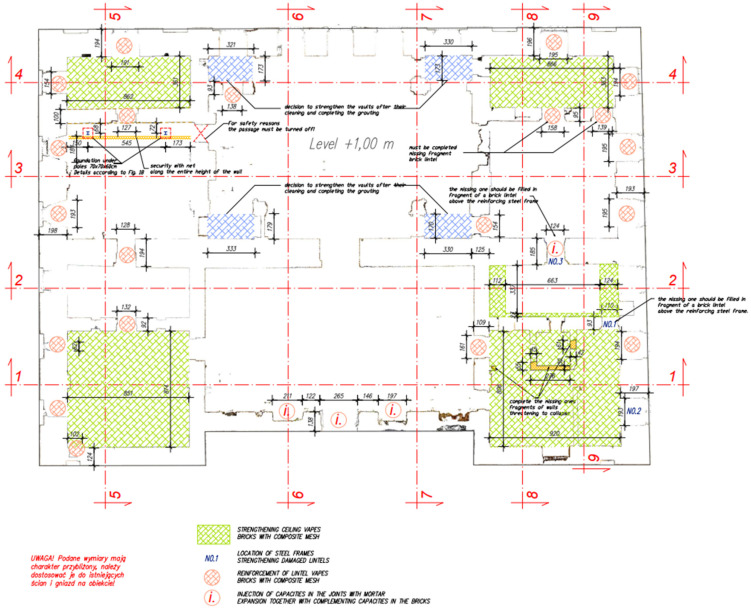
Three-dimensional scanning of the building at a height of +1.0 m above the ground floor (own study and [23]).

The inspiration to take up the topic presented in the article is very frequent abandonment of fragments of historic fortifications in a state of advanced degradation and, thus, exposing them to further destructive influence of the environment [14]. In such situations, it becomes necessary to “prepare” them for further use in the above-mentioned environment while ensuring the required level of reliability [18,19,20]. One example of such structures is the remains of a medieval castle (Figure 1, Figure 2 and Figure 3). In the past, it was the seat of the commander of the Order of St. John. It was built in the years of 1545–1564, replacing the wooden knight’s manor that existed there in the years of 1426–1429. The castle in Słońsk has been expanded and rebuilt many times since its construction. In 1652, it was burned down by the Swedes, and its last expansion took place in 1783 (Figure 1a). Due to a devastating fire that took place in 1975, the facility became a ruin left for over 45 years, threatening a construction disaster. Because it is a monument with high tourist potential, closely related to the rich history of Brandenburg, the Netherlands and Europe, it was decided to preserve it for future generations in its current state (Figure 1b,c).

The medieval walls of the original structure of the facility have survived to this day, and it was decided to secure them. Already in 2018, the technical condition of the castle’s masonry structure was considered disastrous. The threat here was the exposed slender walls 19 m high without stiffening in the form of vaults, wooden ceilings, and a roof. Chimney walls were particularly at risk of losing stability, while the remains of the vaults contained a large layer of rubble and vegetation growing on them (Figure 2). In 2021, the building was protected with a temporary wooden roof and covered with roofing felt, which resulted in the quick drying of bricks and mortars with intensive ventilation caused by the lack of windows and doors. The remains of the vaults, like the rest of the building, were on the verge of a construction disaster. The above-mentioned loss of wooden ceilings, vaults, and large fragments of walls significantly weakened the stiffness of the remaining structure of the building, which was due to the walls being left unprotected for the last 45 years. The facility required urgent protection and reinforcement of walls and lintels, as well as reinforcements and additions to the vaults. Carrying out this work turned out to be an extremely difficult and dangerous activity due to the additional threat associated with the not fully recognized and heavily debris-covered surfaces of the structure, which already lost its character as a building. Debris was lying on the ground floor and on the remains of the vaults, which made it very difficult to inventory places that were then inaccessible, hence scanning the facility [23] turned out to be very helpful as it did not have archival documentation (Figure 3).

By the decision of the monument protection services, the facility was to remain in ruins and intended for sightseeing, without supplementing or rebuilding the deformed structures. The owner of the castle obtained consent only to protect the damaged structures against construction disasters and eliminate the threat to visitors, which turned out to be difficult to implement due to the fact that the standard conditions [18] would not be met both in relation to the requirements for modern building materials and the stability conditions. As mentioned above, a helpful solution was to scan the entire facility to determine the extent of deformation of the remains of its walls and vaults. The first very difficult stage was the removal of rubble from both the ground floor of the castle and its first aboveground floor where the vaults and their fragments remained due to the threat posed by the deformed and heavily strained masonry structure. The benefit of carrying out the above-mentioned activities included not only cleaning up the rubble but also recovering approximately 70% of valuable historical bricks, which were used to supplement and strengthen the structures of walls and vaults. Defective and weakened historic ceramic bricks with a tendency to delaminate, peel, and crumble were replaced with other bricks coming from elements that no longer existed here, and the bricks themselves were preserved in good condition, placed on a sand-lime mortar.

**Figure 4:**
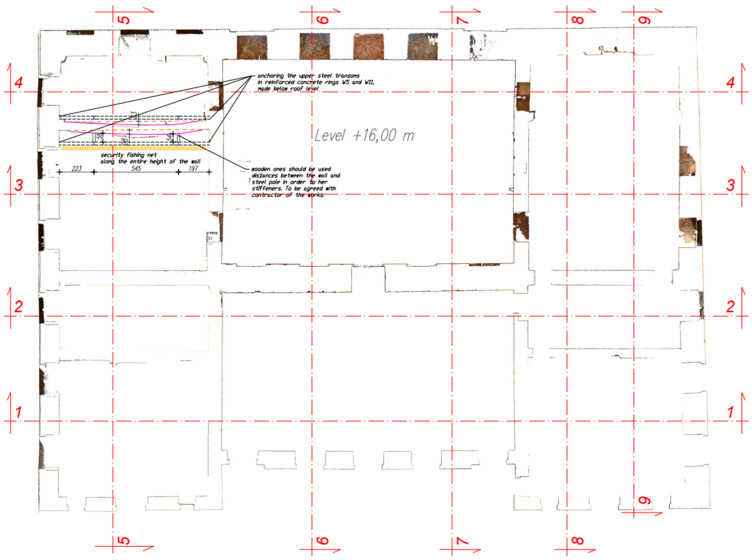
Three-dimensional scanning of the building at a height of +16.0 m above the ground floor (own study and [23]).

In Figure 3, the green color indicates the vaults that remained in the structure of the building in whole or in part, while in Figure 4, the deformed chimney wall that tilted the most from the vertical is marked. 

Testing bricks or other materials cannot be an end in itself when it comes to an existing and still used facility.

The brick itself is only part of the knowledge of the technical value of the masonry structure it creates. Low parameters of individual bricks may disqualify such a structure as a whole or have no major impact on it. Only the assessment of walls in facilities exposed to the negative impact of the external environment, which is a highly unfavorable interference for them, allows for a comprehensive assessment of the tested material, hence the research and analyses should assess the degree of wear of masonry elements and the possible threats this wear may pose. The heterogeneity of the bricks may disqualify the wall as a structure, but the distribution of stresses in the walls at an angle of 60° with the simultaneous elimination of concentrated loads and their considerable thickness (60–200 cm), as well as a dense network of vertical stiffeners, may also allow for further exploitation of the ruin after the introduction of an additional external structure (ensuring the stability of the walls and can be dismantled at any time) and, at the same time, not reducing the authenticity of the monument. Verification calculations for walls with a height of 19 m showed that the compressive stresses in the wall should not exceed the permissible values, even for bricks and (mortar) with a strength of 5 MPa and (1 MPa), and may even be lower by 15%. For 15 MPa class bricks, this reserve would be approximately 60%.

The conservation covered the vaults and walls but only on the surfaces where the bricks lost their structure. In order to strengthen, i.e., harden, the bricks and mortars in the masonry elements, it was proposed to introduce the silicate preparation Primer Hydro SF/Silikatfestiger into their structure, (art. no. 1072, Remmers) which is a liquid, mineral primer with a strong bonding effect. It is a colorless preparation that creates a structure permeable to water vapor, which structurally strengthens from 4 to 8 N/mm² by filling pores and small cracks with silica with a pH of approximately 11.5. It was applied by spraying three times until the substrate was fully saturated with it.

## 3. Review of the Literature

**Figure 5:**
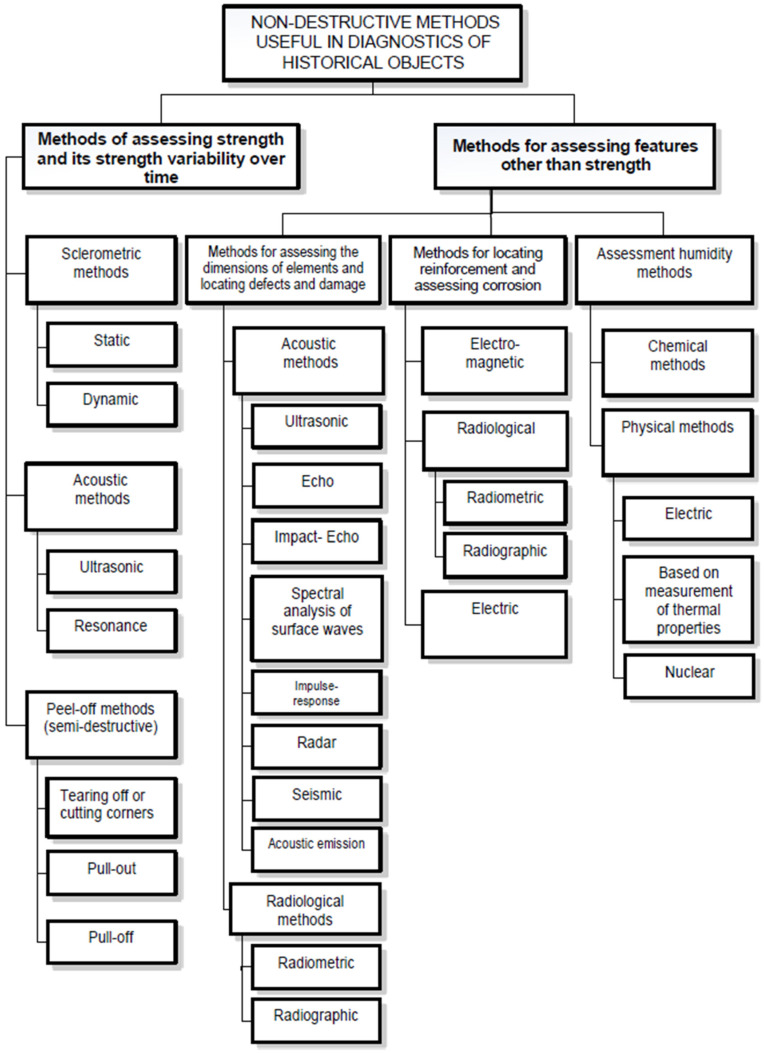
Nondestructive methods useful in diagnostics of historical structures [4,5,6,7,8,10].

In the second half of the 20th century, the Schmidt sclerometer was first utilized to test building ceramics. This tool is still being tested to establish a close relationship between the number of rebounds (an assessment of impact energy) and the compressive strength of bricks and mortars. Although Ernst Schmidt, who patented the device in 1948, originally designed it to assess concrete structures within the measurement range of 10 to 70 N/mm², its application has expanded over time. Currently, work on the application of the above-mentioned instrument for strength testing of materials other than concrete is still based on its original purpose dedicated to concrete. An assessment of the effectiveness of these studies and analyses can be found in refs. [3,4,5,6,7,8,9]. They also constitute a theoretical and practical basis for “transferring” these techniques to other types of materials. Visual inspection should be an integral and basic element of the diagnostics of construction materials and products, as well as entire structures, which will initiate their further treatment. As mentioned above, there are a number of nondestructive methods for diagnosing historical buildings, dedicated mainly to materials used today, i.e., concrete, steel, and wood, which will not always be widely available or fully useful in specific situations. In Figure 5, the authors showcase the aforementioned continuously improved methods. These techniques are designed not only to assess the strength characteristics of elements and structures but also to detect hidden defects, especially in hard-to-reach areas. These defects can significantly impact the durability and safety of both the structures and their surroundings [4,6,7,8,10,11,24]. Knowledge of the location and size of such defects will allow for creating a picture of the damage and, thus, influence the accuracy of subsequent decisions and proceedings.

Already in the 1970s, Prof. Leonard Runkiewicz wrote about the imperfections of sclerometric tests of masonry structures. In his publication [25], Runkiewicz stated that his own research and the analysis of works [26,27,28,29] only indicated certain usefulness of N- and L-type Schmidt hammers for assessing the current strength of built-in bricks. He proposed his own, quite strict correlation between the strength and the number of rebounds of the N-type Schmidt sclerometer, expressed by Formula (1):

*R_c_* = 0.305*L*^2^ − 11.42*L* + 131.6 MPa, (1)

where *L*—number of rebounds.

In work [25], empirical dependencies for ceramic bricks were determined by Formula (2):

*R_c_* = 0.203*L_s_*^2^ − 13*L_s_* + 212.7 MPa, (2)

where *L_s_*—reduced number of rebounds depending on the tension in the wall.

*L_s_* = *mL*

where *m* = 1.00–0.75—coefficient at stresses from 0.0 to 2.0 MPa.

The authors of the above article [25] stated in the summary that Schmidt hammers in testing historic buildings made of bricks could only be used to estimate strength [30]. When testing demolition bricks, due to the influence of small cracks and microcracks, the rebound numbers were reduced by approximately 20–30% compared with new bricks. Tests carried out on bricks by authors of [25] in historic buildings confirmed that small scratches and microcracks have a significant impact on the rebound numbers depending on their strength. It was found that there is a weak relationship between the surface hardness of the old brick and its compressive strength [25].

Nondestructive testing of building ceramics is still a topic of interest for researchers and engineers dealing with historical and contemporary structures, who strive to improve these methods.

The topic of nondestructive and low-destructive testing was discussed by Dawid Łątka in [31,32]. The author developed a unique correlation curve for determining the compressive strength of bricks using the Silver Schmidt sclerometer. This curve considers existing recommended curves for the mechanical version of sclerometers, with the goal of diagnosing masonry structures built to high workmanship standards, such as viaducts and bridges. So far, the most frequently used tool dedicated to quick and noninvasive assessment of structures is a standard sclerometer (Schmidt hammer), but mainly in the diagnosis of concrete structures [33]. Sclerometric tests using the Silver Schmidt electronic hammer enable verification of the homogeneity of the bricks used during the construction of the structure and a preliminary estimate of their compressive strength. Sclerometric tests carried out on brick structures with higher standards of workmanship and the quality of the material used are characterized by a smaller spread of results, which has a beneficial impact on the accuracy of estimating the compressive strength of the wall. There are several documents describing the sequence of proceedings for the sclerometric procedure, mainly regarding the testing of concrete structures, such as the American standard ASTM C805 [34], Polish PN-EN 12504-2 and others [33,35], or the Chinese JGJ/T 23-2011 [24]. So far, no standards have been developed for testing masonry structures, but RILEM Instruction TC 127-MS.D.2 [36] is available, describing a procedure for masonry structures that differs significantly from the one described for concrete structures. Inconsistent results may arise from the lack of standardized testing protocols for masonry structures. A standardized testing procedure must be created and followed to guarantee data accuracy and comparability. The main difference lies in the repeated measurement at a single point, as opposed to concrete testing where measurements are taken at intervals of 20–25 mm. Selecting the test site is crucial to ensure the following conditions: the tested brick, as well as the surrounding bricks and mortar, must be free of cracks and dry. Tests are not performed on edge bricks. Before starting the actual measurement, the hammer pin is placed perpendicularly to a clean and smooth surface and 3–4 strokes are made to better seat the pin head. Then, without removing the pin from the surface, 10 strokes are performed, and the resulting rebound R values are recorded. From these values, the five highest readings are selected. The average value of these selected rebounds, along with the standard deviation, constitutes the result for the given measurement location. Conversion of the obtained results into the compressive strength of bricks is only possible if a minimum of four destructive tests are carried out simultaneously. The number of tests carried out within one area of the structure may depend on the variability of the results obtained during the test, hence a greater number of measurements are required. The outcomes’ variability is not the only factor determining the number of tests. Adhering to a specified testing process should ensure the consistency and dependability of the results. In construction practice, tests are most often carried out according to the rules adopted for concrete structures [37]. 

One of the first publications devoted to the use of a sclerometer to estimate the compressive strength of bricks was the above-mentioned study by Olek et al. [27]. Unlike the current methodology of testing whole bricks, the past standard provided for determining the *f_B_* value on a sample made of two halves of a brick bonded together with cement mortar. The error estimated in the studies was 34%. 

Conversion curve equation according to [7], Formula (3) for previous (information above):

*f_B_*(*R*) = 0.031*R*^2^ − 1.164*R* + 13.418 (3)

The results of research on historical bricks (18th and 19th century) and those manufactured today with the methods used in the past (hand-formed bricks) were published by Egermann [38]. Based on the correlation between the compressive strength of bricks determined according to German standards and the rebound number recorded with the Schmidt hammer, he proposed his own conversion curve. This curve’s abscissa represents the average rebound number R value, determined for the X direction (measurement on the headers) and the Z direction (measurement on the stretchers).

Conversion curve equation according to [38], Formula (4):

*f_B_*(*R*) = 0.04*R_xz_*^2^ − 0.55*R_xz_* + 13.6 (4)

In these tests, a difference was recorded in the readings taken on headers and stretchers, which is related, among other things, to the anisotropic properties of ceramics.

R. Schrank recorded rebound numbers for the 19th-entury bricks in walls made of solid ceramic bricks in lime mortar [37]. He also determined the strength of bricks on the basis of destructive tests according to the DIN 105 standard. The same methods of increasing the confidence level are used in the sclerometric assessment of concrete strength when it is not possible to calibrate the base curve measured in destructive tests.

Conversion curve equation according to [37], Formulas (5) and (6):

*f_B_*(*R*) = 0.00103*R*^3^ − 0.058*R*^2^ + *R* (5)

*f_B_*(*R*) = (0.00103*R*^3^ − 0.058*R*^2^ + *R*)/1.4 (6)

The conversion function developed by Brozovski [39] was based on the results of the research in which a light-type mechanical Schmidt hammer was used. Nondestructive testing was carried out in accordance with EN 12504-2 [33] and CSN 731373 [40] standards, and the compressive strength was determined in accordance with EN 772-1 [41]. Two types of solid ceramic bricks with different dimensions were used, 29 × 14 × 6.5 (cm) and 25 × 12 × 6.5 (cm). The curves were developed for an LB hammer with a rounded shank (dedicated specifically to the diagnosis of masonry structures). It is noteworthy that during the tests with the LB hammer, a determination coefficient *R*^2^ value of 0.956 was obtained, whereas for the L hammer, it was only 0.756. This suggests the greater efficacy of the LB hammer in diagnosing masonry structures.

Tests conducted by Roknuzzaman [42] allowed for the development of two relationships dedicated to testing bricks in a horizontal position. Both curves were characterized by a high value of the *R*^2^ coefficient, i.e., 0.96 and 0.92.

To summarize the content of the above provisions, it should be stated that a uniform method for assessing the compressive strength of bricks in situ has not been developed so far. All the aforementioned attempts could only approximate the strength of the bricks within varying degrees of error. This could be significant for structures already under stress, even if they appear massive. However, there is a fundamental problem here when the research concerns the assessment of wall parameters (strength and homogeneity of wall elements) in larger areas. Their results can only be considered highly approximate due to the scope (not only the method), but they nevertheless relatively indicate the precise differences in the examined wall surfaces and their structures. In situ tests should always be verified by destructive tests, which will not always be possible due to the limited number of samples to be selectively taken if such a situation is possible at all.

## 4. Materials and Methods

### 4.1. Materials

The supporting structure of the castle ruins currently consists of walls and vaults made of solid ceramic bricks with a strength ranging between ~5 and 15 MPa, made of sand and lime mortars, the strength of which was set at 0.5 to 1.0 MPa. Brick is the basic building material of walls here. It was highly degraded biologically and erosively as a result of repeated cycles of strong dampness and drying, as well as by aeolian factors where solid particles carried by the wind caused abrasion of its external surfaces [6,24]. During visual inspection of the walls and vaults, no whitening or salt efflorescence was found, which indicates that the facility was not strengthened or repaired using cement-based materials. Locally, biodegradation was found, mainly on the upper surfaces of the vaults, as a result of the penetration of the roots of the intensively growing vegetation there. However, in the capillaries, there was no water rising from the ground. The main causes of moisture were climatic factors (precipitation, wind, temperature) and the lack of protection of the facility from above. The authors of this article decided to carry out nondestructive tests of the walls in situ and t collect samples of the material for laboratory tests. It was also decided that dirty places would not be cleaned unless the existing coatings were harmful to building ceramics; however, it was considered necessary to carry out biocidal treatments on walls and vaults after removing organic materials and vegetation from them. During the tests, no harmful coatings were found on the bricks, and only those surfaces that were intended for subsequent strengthening were cleaned.

### 4.2. Methods

#### 4.2.1. Testing the Strength of Bricks

**Figure 6:**
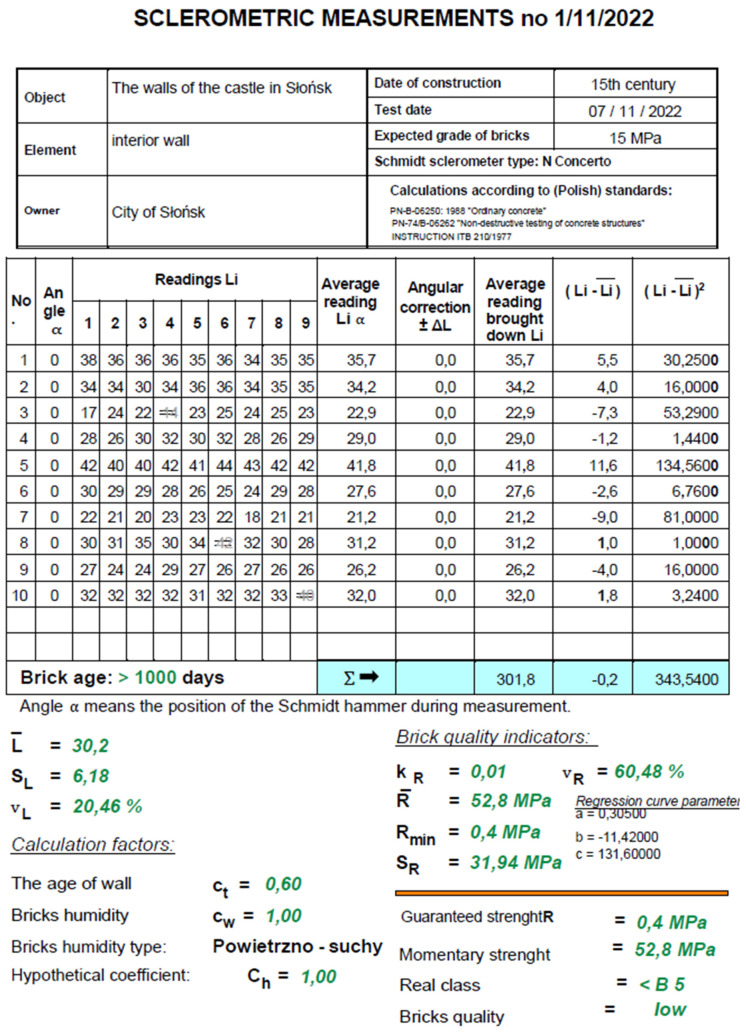
Brick test results obtained with an N-type sclerometer (own study).

**Figure 7:**
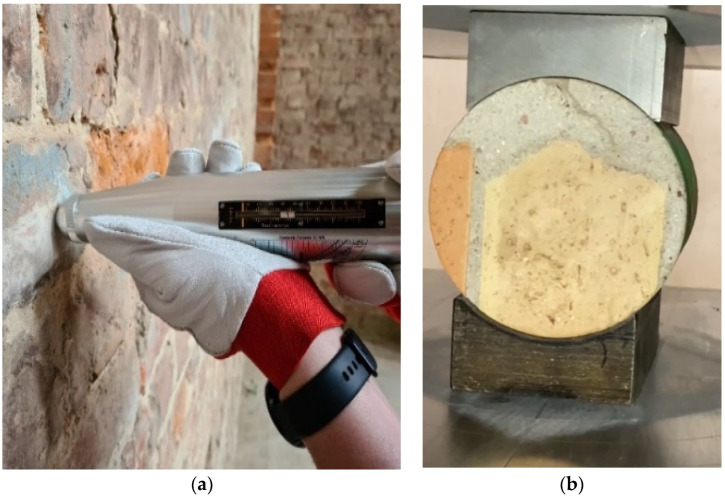
(**a**) Testing the compressive strength of bricks using an N-type sclerometer; view of a sample (**b**) (own study).

As written above, part of the material tests were carried out directly on the site. First of all, they concerned the evaluation of the strength of the embedded bricks using a Schmidt sclerometer (Figure 6 and Figure 7). The findings were compared with the results of destructive tests performed on samples taken at several locations overlapping with the locations of the nondestructive tests. Sclerometric tests confirmed the high structural and strength inhomogeneity of the bricks, which was a further impediment to carrying out subsequent structural protection. The strength of the bricks, determined by the nondestructive method, was determined based on the correlation between their strength and the number of sclerometer reflections using the regression curve formula, among others, for the “N” type hammer, developed by Prof. Leonard Runkiewicz and Eng. Wieslaw Rodzik [25]. Therefore, the compressive strength values obtained this way were considered only illustrative due to the considerable scatter of results, the scale of the object size, the heterogeneity of the material, and the inaccuracy of the testing methods. They did not give the expected unambiguous answer about the current state of the remains of the castle walls, so it was decided to leave the structure of the object unchanged and propose another form of protection. 

Core drillings were taken and then tested in a strength press. The results are given in Table 1.

#### 4.2.2. Testing the Degree of Moisture of Bricks and Salt Content

The subsequent tests were carried out to assess the level of moisture and salinity of the masonry structures.

A commonly used measure of moisture content in building partitions, including walls made of ceramic bricks, is their mass (absolute) humidity. It is described by the percentage ratio of the weight of water contained in the material to its dry weight, as shown in Formula (7).

(7) $$w_{m}=\frac{m_{w}-m_{s}}{m_{s}}100\%$$

where

*w_m_*—mass moisture, w %
*m_w_*—wet weight of wood (sample), in g (kg)
*m_s_*—mass of wood (sample) dried to solid mass, in g (kg)

Mass moisture measurement was performed using the Protimeter MMS2 meter (Protimeter, Crown Industrial Estate Priorswood Road Taunton TA2 8QY, UK (Figure 7). These tests were verified using the laboratory dry-oven test method. The results are presented in Table 2 [43].

**Figure 8:**
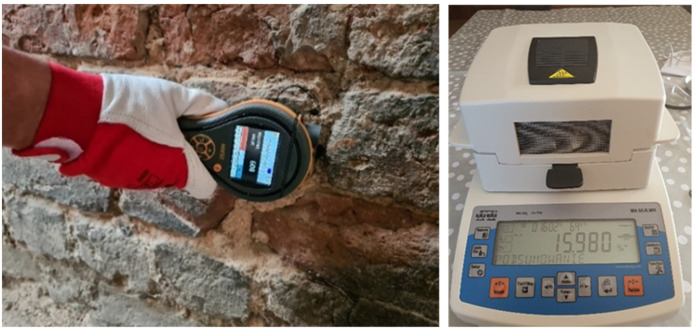
Assessment of the moisture level of walls using the radio and laboratory dry-oven test method (photos by the authors).

**Figure 9:**
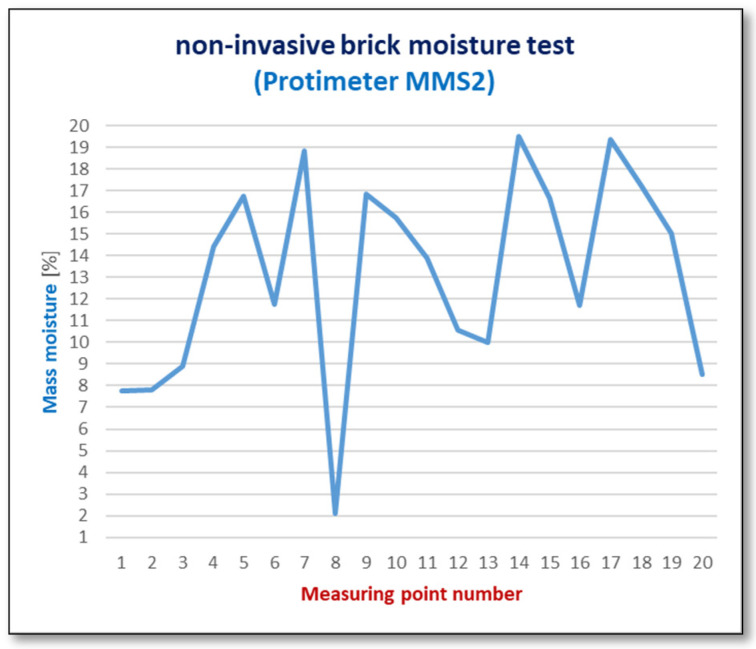
Results of in situ mass moisture measurements using a meter (measurement depth up to 20 mm).

The moisture content of the bricks and the salt content in them were measured in a total of 50 samples. Only three test results showing the extremes of the measured quantities are included in Table 2, while Figure 8 and Figure 9 (for its readability only) includes a graph showing the measurement results obtained at 20 measurement points. Accordingly, the salt content of samples 1, 2, 3 (according to Table 2) was determined.

The graph in Figure 8 shows significant differences in the moisture content in bricks at different measurement points. Until the roofing over the building structure was completed, the walls and vaults showed a very high level of moisture, so they were considered wet. 

The moisture distribution on the wall surfaces was not uniform. The highest moisture levels were observed in the upper parts of the walls where they directly absorbed atmospheric precipitation. Conversely, the lower parts exhibited the least dampness, with a moisture content of less than 6% (7). The roof made in the spring cut off the rainwater supply, and in the summer, it led to their rapid drying, which was intensified by the strong ventilation of the building without windows and doors. This stabilized and evened out the moisture content in the walls to a level not exceeding *w_m_* = 6% (7). However, this situation had an impact on the condition of the heavily damp bricks, which partially lost their compact structure, reducing their active load-bearing cross-sections. The process of natural drying of the wall will continue for several years and it is recommended to continue this method of drying the facility.

**Figure 10:**
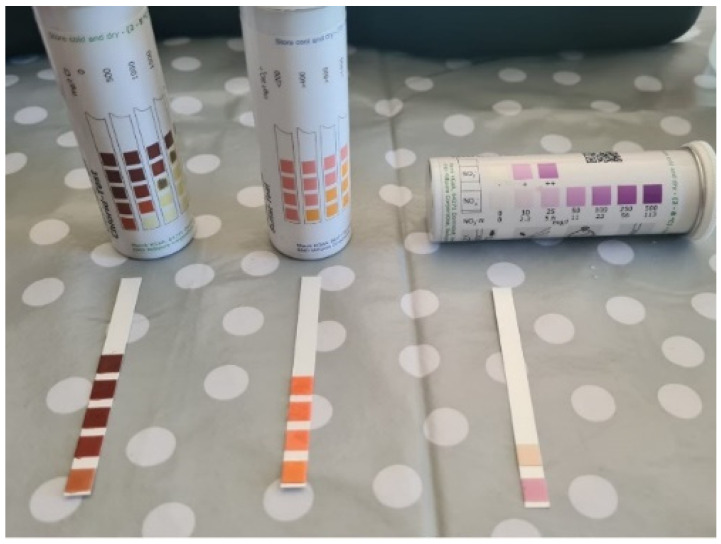
Assessment of salt content carried out on 20 samples taken from various places in the building (photo by authors).

As part of the tests carried out, the level of salt content was determined using the chemical indicator method (Figure 10), the test results are presented in Table 3.

Since the level of nitrates, sulphates, and chlorides was low, these compounds were excluded as the cause of the damage, which confirms the previously stated thesis that the direct cause was the variability of weather conditions and the lack of a roof over the facility.

Another problem and threat to the ruins of the building, apart from the heterogeneity of the wall structure, was the deformation of the high 19 m walls in three directions. The deflection of walls from the vertical (including those in the shape of an arc) reached up to 40 cm and, locally, even up to 1.0 m. 

It was useful to conduct in situ and laboratory tests to assess the condition of the entire structure.

**Figure 11:**
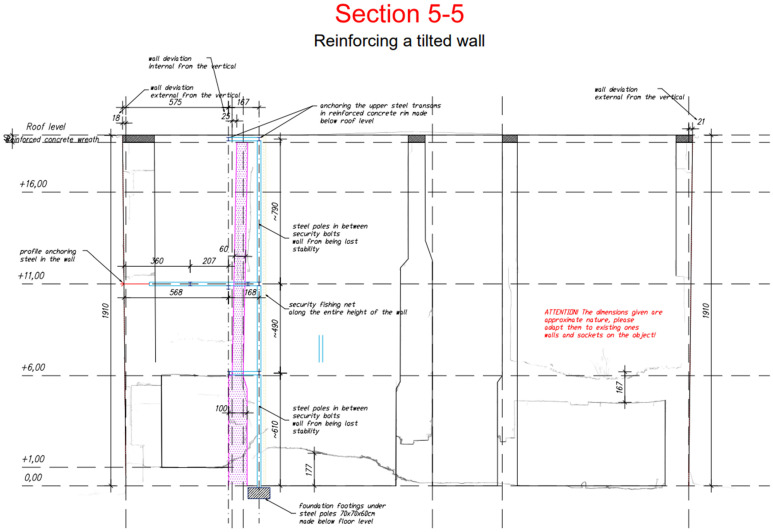
Cross-section through the chimney wall and its front view (own study).

The authors of this article emphasize the importance of material research necessary to maintain the remains of historic buildings that are in a state of technical and functional ruin. The ongoing research of the medieval castle in Słońsk is intended to serve both the preservation of the assessed materials and its remaining ruins for the purposes of further historical searches, archaeological exploration, and public access, constantly increasing knowledge about them. Within the framework of this article, the case of preservation of the remains of the chimney wall located in the northern corner of the building (Figure 5 and Figure 11) is selectively described, bearing in mind the incompletely recognized parameters of the masonry elements due to their heterogeneity in such a sizable volume of the building (about 22,500 m^3^). Attempting any reinforcement, additions, or repointing of the object and its elements would lead to a reduction of its historic character. Therefore, the results of the research provided a partial answer to the issue of further possible although risky treatment. This is how material and environmental research should be directed, and it should not be an end in itself.

The wall in question, intact, was to remain in the object as a witness to the older and recent history of the castle, depicting the turn of its fate to the present day, and the results of the material tests carried out were to assess the possibility of allowing such exploitation. The investigated case is not a duplication of similar standard research but a way of using it for practical as well as research purposes for a specific object, which, thanks to it, was admitted to exploitation bearing in mind the danger that may be posed by the incomplete building materials that make it up. The main objective of this research was to establish the parameters of medieval materials degraded by being left in an environment to which they were not adapted. Their age, fire, and exposure to the external environment without any protection contributed to bringing them to such a state. In addition, the castle’s vaults were subjected to loads of rubble, organic embankment (soil), and vegetation whose roots and moisture had a very negative impact on the structure of the walls and the bricks and mortar themselves.

As part of the analysis and calculations carried out, it was determined that in the extreme case, even after securing the wall structures, there is a possibility of their damage during further operation, including in the presence of tourists. In view of the above, such a state of danger was provided for in the construction solutions. It was proposed to make an independent structure to protect the surroundings from the effects of possible dehiscence and fall of loose bricks and even whole fragments of the wall while respecting the authenticity of the monument. The research and subsequent analysis of the structure in a highly deformed state will allow to keep the monuments—ruins—in the current authentic state for them, not meeting the criteria of bearing capacity and stability required by current regulations and standards [18].

The idea behind this solution was to capture the damaged wall at three levels and protect it against loss of stability by anchoring it to a wall where stability and load-bearing capacity were beyond doubt. The project assumed the possibility of fragments of the wall breaking off in the highest part of the wall and the possibility of them falling to the lowest level, hence the designed steel structure was secured with a steel mesh. This solution took into account the low-strength class of bricks and mortars, their deep losses, and the lack of proper bonds between the masonry elements. In the proposed solution, platforms made of 25 mm thick OSB (Oriented Stand Board) boards were introduced on two levels, the task of which was to dampen the impact of falling debris on two levels, assuming the possibility of their subsequent destruction (starting from the first and then the second platform) in the event of the disaster of the wall in question. The principle of local strengthening of the above-mentioned chimney wall is shown in Figure 11, which was to involve injecting gaps in the joints between the bricks and strengthening the cracked and bulging parts of the walls with composite meshes (FRCM). The use of carbon meshes applied on mineral matrices did not take away from the authentic appearance of the monument because, in the past, its walls were entirely covered with plaster.

**Figure 12:**
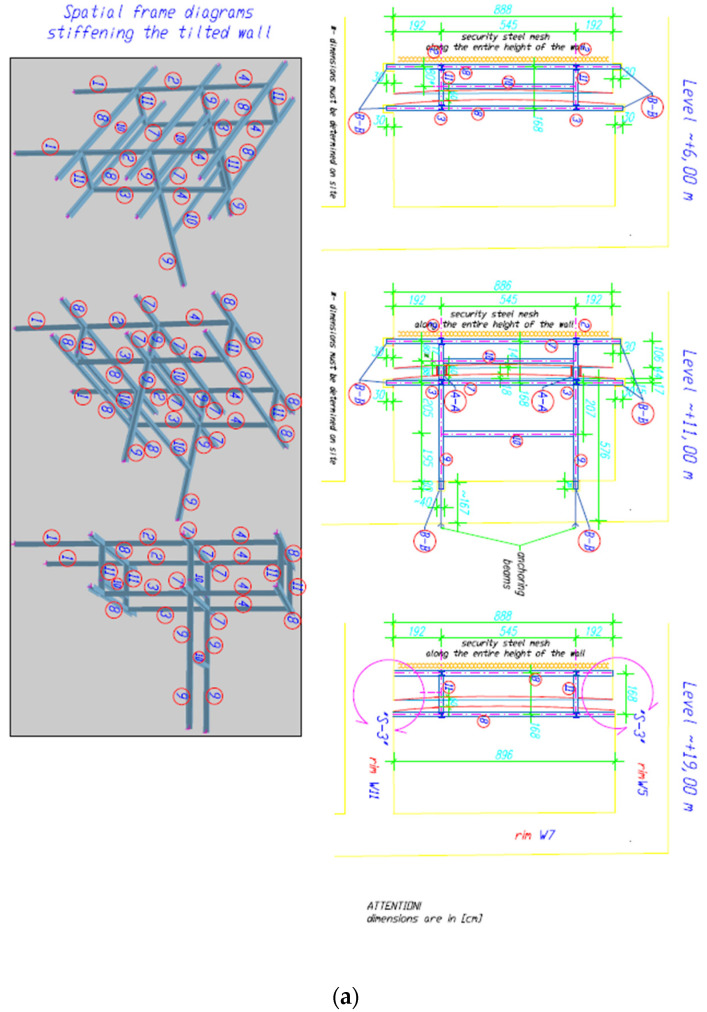

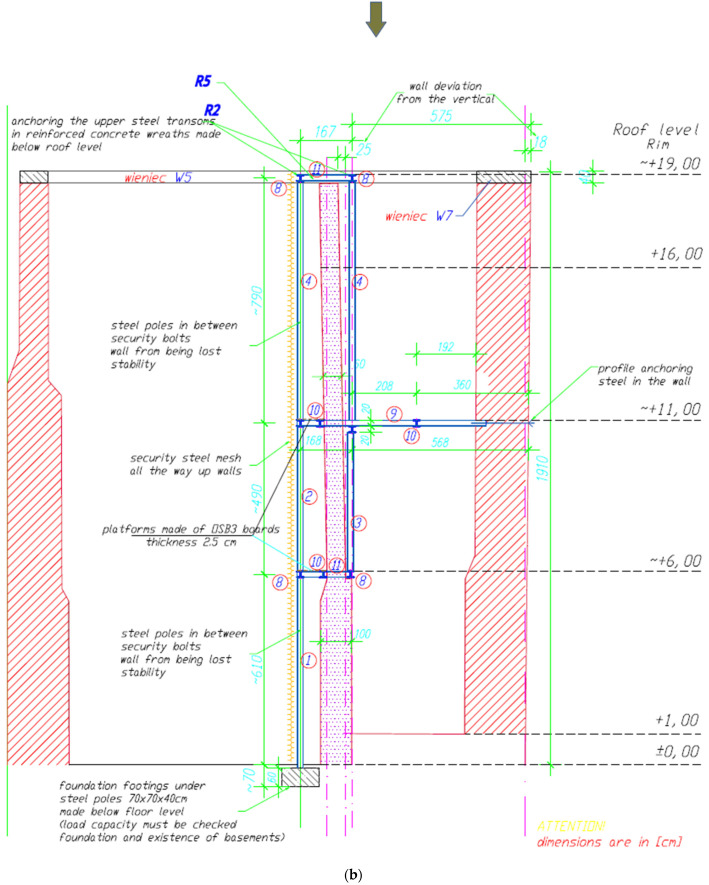
Diagram of an independent structure strengthening the chimney wall (**a**) frames, (**b**) cross section (own study).

**Figure 13:**
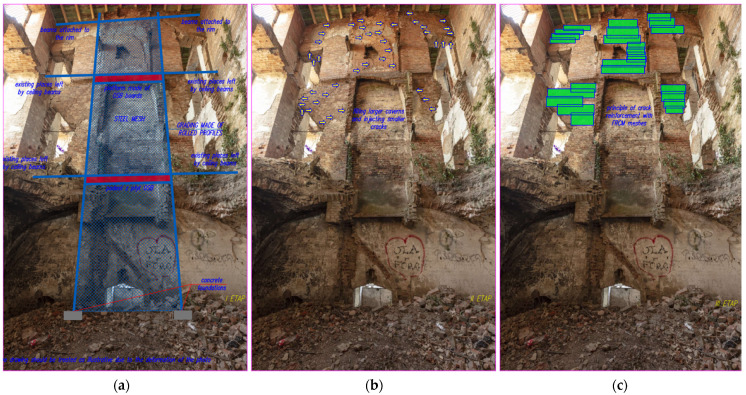
Proposal for strengthening the chimney wall: (**a**) independent steel structure (two platforms intended to transfer the load from falling debris are marked in red); (**b**) places where bricks are joined by the injection of joints (yellow); (**c**) places where strengthening composite meshes (FRCM) are applied—green (photos by authors).

The structure described above was actually made with minor modifications, adapting it to the possibility of installation in the immediate vicinity of the deformed walls. Its final and implemented version is shown in Figure 12 and Figure 13. Currently, this facility is partially open to the public.

As part of the measures available to the current owner of the building, the vaulted lintels were also secured using a method similar to that described above, i.e., without any rebuilding or strengthening. The aim of such actions was to limit the loss of the structure’s authenticity as much as possible.

**Figure 14:**
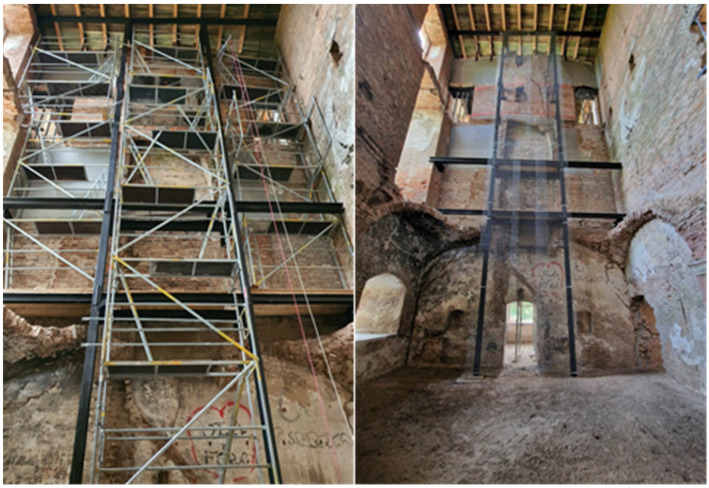
Current view of the strengthened wall and the strengthening structure (photos by authors).

**Figure 15:**
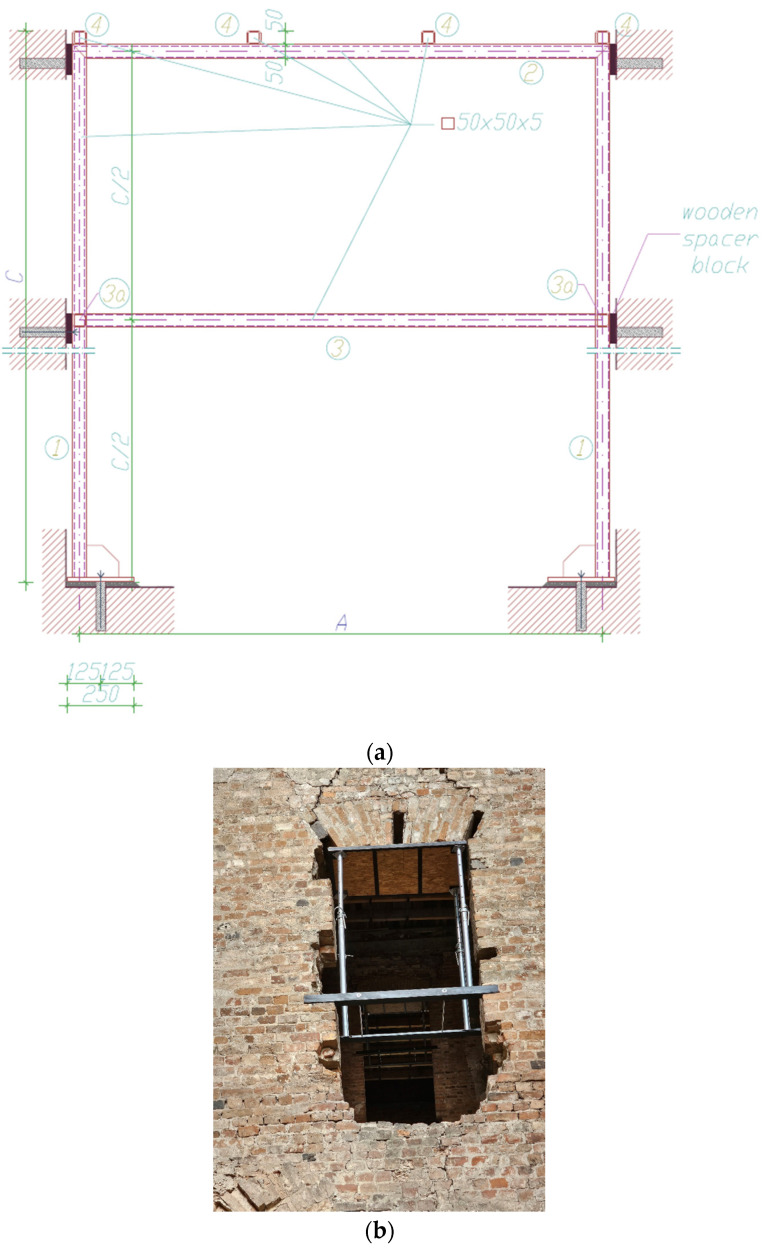
Securing the arches of openings in walls: (**a**) design; (**b**) execution (own study).

**Figure 16:**
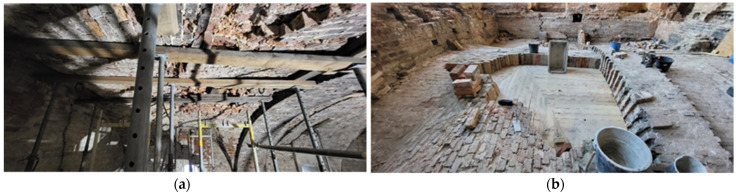
Fragments of the vaults above the ground floor of the castle: (**a**) view of the damaged bricks from below; (**b**) filling in (photos by authors).

As a result of covering the ruins with a temporary roof, the facility began to dry out quickly, which had an impact on the building ceramics, especially the vaults, which, after removing unnecessary ballast and cleaning, turned out to be severely deformed structures with local, but deep, defects. Moreover, as a result of drying, the bricks lost their structure and their bottom surfaces became detached to a depth of up to half of their thickness. Therefore, the planned method of strengthening them on both sides with composite meshes on mineral mortars could only be applied locally and only in less damaged structures. Some of the vaults required additions (Figure 14, Figure 15 and Figure 16). This was achieved by reproducing the original, using the recovered bricks.

As part of the rescue and security works, biological contamination was neutralized with biocides in the form of an alkonium-free chloride solution (pH: approx. 7.5) dedicated to removing algae, fungi, lichen, and moss spores from the surfaces of the mineral building materials. This measure was used in places where organic materials had previously accumulated on the vaults and the ground floor at their junction with the walls, after their prior removal. Agents based on silicic acid esters were used to consolidate the weakened structure of the bricks. In places where the application of strengthening composite materials was planned, the load-bearing capacity and adhesion to the surfaces of ceramic walls and vaults were checked using the pull-off method. The peel strength of the test discs should not have been less than 1.5 MPa.

The most damaged “chimney” wall was strengthened and protected by:

− Preliminary ad hoc stamping on both sides;
− Cleaning the floor of the rooms and setting up scaffolding;
− Construction and assembly of a steel reinforcing structure and equipping it with light platforms on two levels (Figure 10, Figure 11 and Figure 12);
− Placing OSB3 boards on platforms to reduce the effects of the impact of a falling wall fragment—they may break, which will allow the rubble to slide more gently to the lower level and the ground floor;
− Filling larger gaps between vault bricks with expansive mortars based on lime, cement, and trass;
− Introduction of reinforcements on damaged wall surfaces using C-FRCM meshes on inorganic mortars; *
− Removal of temporary stamping structures and scaffolding.

* Damaged wall structures were surface-reinforced with carbon fiber meshes on inorganic mortars based on the Ruredil C-MESCH GOLD 84/84 system (Visbud-Projekt Sp. z o.o. Wrocław, Poland). A layer of inorganic matrix based on Ruredil X Mesh M25 pozzolans (Visbud-Projekt Sp. z o.o. Wrocław, Poland) for masonry substrates with a thickness of 3 mm was applied on the stripped, cleaned, and moistened brick substrate, after prior checking of the adhesion of future composites to the substrate using the pull-off method (the pull-off strength of the discs exceeded 1.5 MPa). In the next stage, the Ruredil X Mesh C10 (Visbud-Projekt Sp. z o.o. Wrocław, Poland) mesh was “embedded” and a second layer of Ruredil X Mesh M25 mortar (Visbud-Projekt Sp. z o.o. Wrocław, Poland), also 3 mm thick, was applied (Figure 11 and Figure 12). One-sided or double-sided reinforcement or strengthening of the vaults was made as described above after injecting the gaps in the joints between the bricks.

## 5. Results

**Figure 17:**
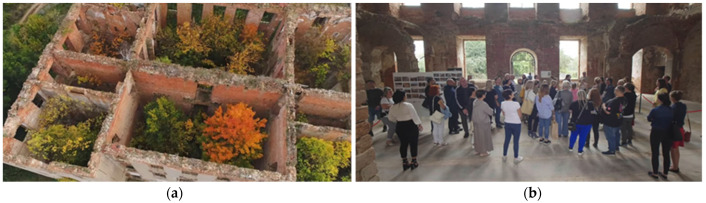
Ruins of the castle in Słońsk: (**a**) initial condition; (**b**) current condition (photos by authors).

A wide range of tests carried out on the facility and, as a result, the proposal and implementation of very low-invasive interventions in the structure of the walls made it possible to stop the degradation processes of this structure and its elements while maintaining them in a visually unchanged (authentic) state. Currently, it has been monitored for 3 years, and at the same time, it has been made available to the public for viewing (Figure 17).

Test methods for valuable structures and building materials should be selected so as to pose the least possible threat to unique, historical structures while maintaining the highest degree of their authenticity (Table 4).

For nondestructive tests of the compressive strength of bricks, Formula (1) for the regression curve of L. Runkiewicz according to [25] was used, which gave values close to the results obtained in destructive tests.

## 6. Discussion and Conclusions

The problem of saving monuments, or more precisely the materials from which they were made, is very complex despite appearances indicating the simplicity of their construction. Often, in addition to the knowledge of the principles of statics and the properties of the materials used, it is also required to take into account the effects of physical processes that may occur inside the partitions. There are no two identical buildings or identical structures or materials, hence any interference with a historical building should take into account not only protecting it against further degradation, or even destruction, but also preserving and exhibiting the solutions used in it, as well as the effects of the solutions proposed in it, which should also be monitored in their future operation. In this article, the authors describe a case that occurs commonly and often leads to the reduction of national heritage resources if such a problem is not noticed and treated appropriately at the right time. More and more often, the so-called permanent ruin is mentioned, i.e., something that not only does not have the same splendor as the original but is already in a state of “technical agony” but still constitutes a valuable cultural, scientific, and historical value. 

This article is a brief summary of the possibilities of dealing with immovable monuments, protected by law, for which the combination of field, laboratory research, and analysis of the structure gives the most feasible picture of their preservation. The authors, using proven and pioneering research methods, developed a model for saving a highly damaged masonry structure, proposing an entirely different approach to preserving historically and scientifically valuable buildings/structures by leaving them unchanged or even damaged. The only change here was the introduction of lightweight, external, low-exposure structures in relation to the original to be protected, ensuring that they remain statically balanced and protected from danger to themselves and their surroundings. There are no methods that will unequivocally be able to assess the properties of embedded materials. This is due to the not yet fully developed testing techniques, the range of their capabilities, and above all, sizable areas (volumes) of the tested objects with heterogeneous structures. Not always and not everywhere will it be possible to successfully implement proper diagnostic techniques and traditional repair methods regardless of the type of material and technology used. This article is, in a sense, a case study; nevertheless, it can be successfully applied to other similar situations where urgent salvage work is required, which, due to the extent of the degradation present or the threat they may pose to themselves and their surroundings, is not possible to implement in the traditional manner. At present, the site, left in a state of safe (permanent) ruin, allows for the research to continue and, at the same time, is open to the public, serving as a tourist attraction. The targeted and ad hoc safeguards applied thanks to the knowledge of its properties allow for historical and archaeological research to continue in it. Since the walls and vaults are still drying out naturally and this process will continue for several more years, along with the changes in the structure of the bricks, monitoring these materials and structures by measuring moisture content and checking for any deformation continues. In such situations, it should not be limited to conducting material tests alone in isolation from their role in the construction of the structures they form, especially in the case of large-scale structures. In such cases, the heterogeneity of the parameters and the state of preservation of the materials—here, bricks and mortar and their interrelationships—determine the strength and stability of what they were used for. The authors of this article constantly carry out monitoring of the masonry structure, taking into account changes in the parameters of bricks and mortars over time due to their drying out after securing the castle with a roof. Such an activity is an excellent testing ground for the change in operating conditions to the already favorable indoor climate and progressive degradation of the materials originally examined, mainly due to their drying out and changes in structure. Such research is still being conducted and the results will be the subject of a separate publication.

The literature review along with the research and practical experience of the authors of the article confirms the statement that there are no universal research methods, especially if they concern centuries-old building materials. In addition to the assessment of physical and strength parameters, there remains the historical aspect of the technology and conditions of their (old buildings) execution. Decisions made on the basis of research carried out contain a certain amount of risk, which is particularly dangerous for unique materials and structures made from them (historical materials). Hence, the results of all tests should be treated as initial material for in-depth analyses, both in terms of static strength assessments and those supported by historical queries. The authors of this article have determined the preliminary parameters of the bricks in terms of their strength, structural integrity, and harmful moisture. The fourth parameter of the research in question was monitoring the facility with a simultaneous analysis of the changing environmental humidity conditions (after roofing the ruins) and their impact on changing the properties of bricks and mortars losing the above-mentioned structural integrity. This condition of the bricks also influenced the results of nondestructive tests of the bricks where their top layer with a loose structure influenced the reading of the size of the Schmidt sclerometer reflections and, thus, lowered the read compressive strength of the bricks. The loose structure of the bricks also eliminated the initially assumed method of strengthening the vaults by applying composite materials to their bottom surfaces. The conducted research facilitated the development and selection of strategies aimed at preserving the original structural integrity and external aesthetics of the building while ensuring the necessary level of reliability. As part of the safety and repair works, three different methods of strengthening and securing the structure were introduced, which was adequate to the scope of the damage and their location. The authors of this article also considered the potential challenges associated with predicting the behavior of the structure, particularly concerning the loss of cohesion in unexamined areas of the walls. The efficacy of the diagnoses and chosen solutions will depend on continued monitoring and observations of the structure over time.

Apart from the scientific and technical aspects, there is also a social aspect. The materials that are the subject of this article (brick, mortar) constitute a structure for which an appropriate role has been designated, and its parameters preserved so far are to ensure the durability and safety of the remains of the facility. The article not only describes a case study but also covers the problem in much greater detail, going beyond the framework of typical laboratory tests and the creation of statistical curves, which are an end in themselves. We are dealing here with a valuable historical object for which the authors of this article accepted responsibility. The article indicates methods of handling severely degraded structures caused by damage to their components, which are periodically exposed to unique loads over a long period of nearly 50 years, which are parallel to the standards used in the case study. This is not a typical load case associated with degraded materials constituting the building structure, and the proposed methods based on commonly used technologies are not conventional. This article shows a way to preserve the existing condition (and even appearance) of materials without using invasive techniques and materials that change their image and character. An inherent element of such tests is static and strength calculations, which are not attached to the article due to their extensiveness. Nevertheless, in situ tests, verified by random laboratory tests, have indicated the possibility of further operation of the damaged facility. The measurement of humidity and the examination of the absorbability of the bricks allowed for a clear conclusion that these were not masonry elements adapted to external exposure without additional protection, i.e., plasters. This conclusion concerned both the external and internal walls of the castle. No two objects are the same even though they could be made of similar materials, by the same work team, and in identical circumstances. Hence, in such cases, there are no universal research methods that can solve every problem. The authors pointed to an unusual form of solving the problem, which may be applicable in other similar cases but only after prior assessment of the degree of degradation of the object and the threat it may pose. As it is written in the article that nondestructive methods of estimating the strength of bricks and mortars are still in the research stage, most such structures are not able to wait until the end of the research and testing stage. What we are dealing with here is a variety of masonry elements in one historic structure at a risk of loss. The research carried out was of an implementation nature, which is currently being verified through observation and further analyses in terms of possible new damage, deformations, and durability of the solutions used. This masonry structure is the target of further interest, observation, and research.

The research carried out allowed for the assessment of the state of preservation of the remains of walls and vaults of a valuable medieval monument with a height of 19 m and a volume of 22,500 m^3^, which consequently saved ~8000 m^3^ of walls and 210 m^2^ of the remains of vaults made of solid ceramic bricks in lime mortar. In this case, the structures preserved in their original state should be considered as the starting research material for further investigations and monitoring of the facility, especially in terms of the effectiveness and durability of the structural bond of the bricks that have lost their cohesion as they dried out due to the cutoff of the “moisture supply”. Continuation of geodetic measurements will allow for the assessment of the stability of the entire masonry structure and, thus, the analysis of its degree of safety as a function of time, which should be helpful in situations that limit the possibility of taking samples for destructive testing or even in situ testing. Nowadays, we hear more and more often about leaving historical objects in the so-called “permanent ruin”, hence the research described in this article may prove very useful when making such risky decisions. When deciding to leave historical objects in their current condition, the European Monument Protection Services refrains from trying to restore their original appearance and function because this would involve interference that would reduce their historical value and authenticity. On the other hand, masonry structures left in this form may not fully meet the safety requirements imposed by currently applicable regulations and standards. Researchers of objects and of building materials should not only analyze and evaluate the obtained research data and calculation results but also develop rescue and repair methods, assuming responsibility for the decisions made and, in such cases, protecting cultural heritage.

## Figures and Tables

**Table 1:** Brick test results.

Sample No.	Sample Weight	Average Height/Length of the Prepared Sample	Cross-Sectional Area	Destructive Force	Compressive Strength
	[kg]	[mm]	[mm^2^]	[kN]	[N/mm^2^]
1	0.867	98/98	7543	37.8	5.0
2	1.387	154/98	7543	83.1	11.2
3	1.02	122/98	7543	39.4	5.3
4	0.795	98/85	7543	37.2	4.9
5	0.946	99/98	7543	40.3	5.5
6	0.835	89/89	7543	42.1	5.4
7	0.963	96/89	7543	60.4	7.2
8	1.234	121/92	7543	41.1	5.6
9	1.097	101/90	7543	68.0	6.1
10	1.125	124/93	7543	38.2	5.1

**Table 2:** Results of moisture level tests for selected extreme cases of brick moisture.

Sample No.	Moisture	Mark	Moisture Meter Reading
1	5.56%	moderately moist	≤650
2	15.98%	wet	~999
3	16.82%	wet	~999

**Table 3:** The determined salt content in the collected brick samples (own study).

Type of Salt	Determined Values (% of Mass)			Compartment Qualifying (% of Mass)	Mark
	Sample No. 1	Sample No. 2	Sample No. 3		
nitrates NO_3_	0.005%	0.005%	-	<0.100	low
sulfur SO_4_	0.2%	0.2%	-	<0.500	low
Chlorides Cl	0%	0%	-	<0.500	low

**Table 4:** Brief description of methods of dealing with the tested object.

Name	Description
1. Preparatory work	⮚ Cutting off the supply of further portions of moisture from the walls of a facility that has not been protected for 50 years (roofing and natural drying time); ⮚ Literature review in the field of research methods used and currently being developed; ⮚ Historical inquiry regarding the past of the monument and changes that have been made to it, including the period of neglect over the last 50 years; ⮚ Preliminary inspection of the entire facility; ⮚ Preparing the remains of the castle structure for inventory—enabling safe access to it. ⮚ Inventory of structures—manually and digitally (3D scanning);
2. Laboratory tests	⮚ Selection and indication of places to take samples of bricks and mortars and then collecting them; ⮚ Destructive compressive testing—bricks and bricks containing mortar fragments—support joints (these bricks were previously subjected to nondestructive testing using a sclerometer to determine the hypothetical coefficient); ⮚ Determining the water content in bricks; ⮚ Determination of salt content in bricks; ⮚ Determination of brick absorption;
3. “In situ” brick testing	⮚ Visual assessment of damage; ⮚ Sclerometric tests of the compressive strength of bricks. ⮚ Assessment of the degree of moisture using a nondestructive method; ⮚ Measurement of erosion losses (including mortars in joints);
4. Analysis of the study results	⮚ Assessment of the strength of individual samples and the dispersion of results. ⮚ Assessment of the size of net section losses in bricks and mortars; ⮚ Assessment of the load-bearing capacity of the wall; ⮚ Selection of a method for structural bonding of bricks heavily degraded by the external environment.
5. Determining the amount of load that may be imposed on existing walls after taking into account significant differences in the load-bearing parameters of the bricks and mortar losses in the joints, as well as taking into account load imperfections.	
6. Developing rescue and repair methods for damaged masonry structures.	

## Data Availability

Data are contained within the article.
